# Plasma Ischemia-Modified Albumin Levels and Dynamic Thiol/Disulfide Balance in Sickle Cell Disease: A Case-Control Study

**DOI:** 10.4274/tjh.2018.0119

**Published:** 2018-11-13

**Authors:** Oğuzhan Özcan, Hüseyin Erdal, Gül İlhan, Damla Demir, Ahmet Burak Gürpınar, Salim Neşelioğlu, Özcan Erel

**Affiliations:** 1 Mustafa Kemal University Faculty of Medicine, Department of Biochemistry, Hatay, Turkey; 2 Mustafa Kemal University Faculty of Medicine, Department of Molecular Biochemistry and Genetics, Hatay, Turkey; 3 Mustafa Kemal University Faculty of Medicine, Department of Internal Medicine, Hatay, Turkey; 4 Tokat State Hospital, Clinic of Biochemistry, Tokat, Turkey; 5 Yıldırım Beyazıt University Faculty of Medicine, Department of Biochemistry, Ankara, Turkey

**Keywords:** Sickle cell disease, Thiol/disulfide homeostasis, Oxidative stress, Ischemia-modified albumin

## Abstract

**Objective:**

Sickle cell disease (SCD), described as a group of inherited blood disorders, affects millions of people throughout the world and is particularly common in the southern part of Turkey. We aimed to determine the relationship between ischemia-modified albumin (IMA) and the dynamic thiol/disulfide balance in SCD.

**Materials and Methods:**

Fifty-four adult SCD patients and 30 healthy controls were included in the study. The 54 adult patients included 30 (56%) males and 24 (44%) females with a mean age of 28.3±8.4 years (minimum-maximum: 18-46 years). Of the 54 patients, 46 had homozygous sickle cell anemia (HbSS) and 8 had sickle/β-thalassemia (HbS/β+-thalassemia). Fasting blood samples were collected. After centrifugation at 1500×g for 10 min, plasma samples were portioned and stored at -80 °C. IMA levels were determined by albumin cobalt binding test, a colorimetric method. Total and native thiols and disulfide were analyzed with a novel spectrophotometric method.

**Results:**

We found significantly lower levels of native thiol (-SH) (284.0±86.3 µmol/L), disulfide levels (14.6±7 µmol/L), and total thiols (-SH + -S-S-) (313.0±89.3 µmol/L) in SCD patients compared to healthy controls (respectively 417.0±54.2, 22.7±11.3, and 462.0±58.7 µmol/L). Plasma albumin levels (34.9±7.9 g/L) were lower and IMA levels (13.6±3.1 g/L) were higher in SCD patients compared to controls (respectively 43.5±3.1 and 8.4±1.6 g/L). Plasma albumin levels were strongly correlated with both plasma native (r=0.853; p=0.0001) and total thiols (r=0.866; p=0.0001).

**Conclusion:**

Decreased plasma native and total thiol levels and increased IMA levels are related to increased oxidative stress and provide an indirect and quick reflection of the oxidative damage in SCD patients.

## Abstract (tr)

**Amaç:**

Kalıtsal kan hastalıklarının bir grubu olarak tanımlanan orak hücre hastalığı (OHH), tüm dünyada milyonlarca insanı etkilemekte ve özellikle Türkiye’nin güneyinde yaygın olarak görülmektedir. OHH hastalarında, iskemi modifiye albümin (İMA) ve dinamik tiyol/disülfit dengesi arasındaki ilişkiyi belirlemeyi amaçladık.

**Gereç ve Yöntemler:**

Çalışmaya, yaş ortalaması 28,3±8,4 (minimum-maksimum: 18-46) olan 30 (%56) erkek ve 24 (%44) kadın olmak üzere 54 yetişkin OHH hastası ve 30 sağlıklı kontrol dahil edildi. Elli dört yetişkin OHH hastanın 46’sında homozigot orak hücre anemisi (HbSS) ve 8’inde orak/β-talasemi (HbS/β+-talasemi) vardır. Hasta ve kontrol gruplarının açlık kan örnekleri toplandı. Plazma örnekleri 1500×g’de 10 dakika santrifüj edildikten sonra porsiyonlandı ve -80 °C’de saklandı. İMA seviyeleri, kolorimetrik bir test olan albümin kobalt bağlanma testi ile belirlendi. Toplam ve serbest tiyoller ve disülfit düzeyleri yeni bir spektrofotmetrik metot ile analiz edildi.

**Bulgular:**

OHH hastalarında sırasıyla serbest tiyol düzeyleri (-SH) (284,0±86,3 µmol/L), disülfit seviyeleri (14,6±7 µmol/L) ve total tiyol (-SH + -SS-) düzeylerinin sağlıklı kontrollerle karşılaştırıldığında anlamlı derecede düşük olduğu bulundu (313,0±89,3 µmol/L) (417,0±54,2; 22,7±11,3; 462,0±58,7 µmol/L). OHH hastalarında kontrol grubu ile karşılaştırıldığında plazma albümin seviyeleri (34,9±7,9 g/L) daha düşük ve İMA düzeyleri (13,6±3,1 g/L) daha yüksek bulundu (sırasıyla 43,5±3,1; 8,4±1,6 g/L). Ayrıca, plazma albümin düzeylerinin, hem serbest tiyoller (r=0,853; p=0,0001) hem de total tiyoller (r=0,866; p=0,0001) ile güçlü bir korelasyona sahip olduğu bulundu.

**Sonuç:**

Azalmış plazma serbest ve total tiyol seviyeleri ve artmış İMA seviyeleri, artmış oksidatif stres ile ilişkilidir ve OHH hastalarında oksidatif hasarın dolaylı ve hızlı bir yansımasını sağlar.

## Introduction

Sickle cell disease (SCD), described as a group of inherited blood disorders, affects millions of people throughout the world and is particularly common in the southern part of Turkey [1]. It is characterized by chronic anemia and painful events mainly related to tissue and organ damage. The primary cause of this disorder is a single DNA base mutation that results in the substitution of valine for glutamine at position 6 in the β-globin chain of hemoglobin [2]. This single base mutation induces the production of abnormal and insoluble hemoglobin S (Hb S), which is accumulated especially in anoxic conditions, leading to erythrocyte sediments by sickling [3]. Interaction of sickle erythrocytes with capillary endothelium initiates ischemic end-organ injury via a cascade of thrombotic, inflammatory, and oxidative insults that is exacerbated during painful vaso-occlusive crises [4]. These interact by changing the imbalance of reactive oxygen species (ROS) and antioxidants, causing oxidative damage to cell structures such as lipids, membranes, proteins, and nucleic acids [5]. The imbalance between free radicals and antioxidants also affects sulfhydryl groups (-SH) of organic sulfur derivatives (thiols, RSH), which are represented by serum proteins, mainly albumin [6], and play crucial roles in redox homeostasis. The increase in oxidative stress leads to imbalance in the reversible formation of dynamic disulfide bonds between protein thiol groups [7]. Increasing evidence indicates the vital roles of dynamic thiol-disulfide homeostasis in the regulation of intracellular enzymatic activity, antioxidant protection, and apoptosis, which are related to the pathogenesis of a variety of diseases including diabetes mellitus [8], cardiovascular diseases [9], and cancer [10]. 

Oxidative stress also causes the reduction of the binding affinity of albumin due to free radical damage to the N-terminal of albumin molecules [11]. This new chemically changed albumin is called ischemia-modified albumin (IMA) and is used as a sensitive biochemical marker of ischemia and oxidative stress originating as a consequence of tissue hypoxia [12]. IMA has been shown to increase in patients with ischemic conditions including acute coronary syndrome, stroke, and chronic liver diseases [13,14,15].

However, there is no study in the literature evaluating dynamic thiol/disulfide hemostasis and IMA levels in patient with SCD. In light of those studies that indicated the role of increased oxidative stress in SCD, we hypothesized that thiol/disulfide hemostasis could be related to the pathogenesis of the disease.

In this study, we aimed to evaluate the relationship between IMA and the dynamic thiol/disulfide balance in sickle cell patients.

## Materials and Methods

### Study and Control Groups

Fifty-four SCD patients, including 30 (56%) males and 24 (44%) females with a mean age of 28.3±8.4 years (minimum-maximum: 18-46 years), in steady-state condition who applied to the hematology department of Mustafa Kemal University Hospital, were enrolled in the study. The control group consisted of 30 age- and sex-matched healthy subjects. Of the 54 patients, 46 had homozygous sickle cell anemia (HbSS) and 8 had sickle/β-thalassemia (HbS/β+-thalassemia).

Informed written consent was obtained from all patients and healthy controls. Demographic data were collected from the hospital information system. Patients with diabetes mellitus, obesity, kidney or liver failure, coronary heart disease, any hematologic disorder other than SCD, and malignancy were excluded from the study, as were smokers, alcohol drinkers, and patients with a recent history of transfusion in the last 3 months. The institutional Ethics Committee of Mustafa Kemal University approved the study protocol (protocol number: 2016/90).

### Specimen Collection

Fasting venous blood samples were collected into vacutainer tubes containing ethylenediaminetetraacetic acid and lithium-heparin from the sickle cell patients and healthy controls and were centrifuged at 1500×g for 10 min within 1 h after sampling. After separation, plasma samples were aliquoted for albumin, total thiol, native thiol, and IMA measurements and stored at -20 °C until the time of assay.

### Measurement of Biochemical Parameters

### Chemicals and Devices Used in the Assays

All reagents and chemicals were purchased from Sigma-Aldrich. Spectrophotometric analysis was performed with a Shimadzu UV-1800 spectrophotometer with a temperature-controlled cuvette holder and an automated analyzer (Architect Plus C8000, Abbott, USA).

### Assay Principle of Thiol/Disulfide Homeostasis Parameters

Native thiol and total thiol levels were assayed with a newly developed spectrophotometric method as described previously by Erel and Neselioglu [7]. Briefly, disulfide bonds were first reduced to form free functional thiol groups using sodium borohydride. The unused sodium borohydride reductant was removed with formaldehyde to prevent reduction of DTNB (5,5’-dithiobis-(2-nitrobenzoic) acid). After the reaction with DTNB, the thiol groups including reduced and native thiol groups were determined spectrophotometrically at 415 nm. Disulfide concentration and disulfide/native thiol percentages were calculated with the following formulas:

Disulfide levels (µmol/L) = (total thiol - native thiol)/2.

Disulfide/native thiol percentage (%) = (disulfide x 100)/native thiol.

Since most of the serum thiols are formed by human serum albumin thiols (HSA-SH, ~80%), we calculated the adjusted total thiol, native thiol, and disulfide levels based on serum albumin concentrations from the following formulas:

Adj. total thiol levels = total thiol (µmol/L)/alb (g/L).

Adj. native thiol levels = native thiol (µmol/L)/alb (g/L).

Adj. disulfide levels = disulfide (µmol/L)/alb (g/L).

### Ischemia-Modified Albumin and Plasma Albumin Levels

IMA levels were determined by albumin cobalt binding test, a rapid colorimetric method developed by Bar-Or et al. [16]. The method is based on the binding ability of reduced cobalt ions (Co^2+^) of HSA due to ischemia. Briefly, a known amount of exogenous Co (CoCl_2_) was added to serum samples. Albumin, which is altered as a result of ischemic processes, binds to the Co(II) to a far lesser extent and the excess (unbound) amount of Co^2+^ forms a colored complex with dithiothreitol, which is measured spectrophotometrically at 480 nm. Plasma albumin levels were measured in a calibrated and well-controlled autoanalyzer using the bromocresol green method (Architect Plus C8000, Abbott, USA).

### Statistical Analysis

Analysis of study data was carried out using MedCalc for Windows, version 15.8. Data normality was examined with the Kolmogorov-Smirnov test. Qualitative data were evaluated using the chi-square test and quantitative data were tested utilizing the Kruskal-Wallis and Mann-Whitney U tests. Correlations were assessed using the Spearman test. Values of p<0.05 were regarded as statistically significant.

## Results

A total of 54 adult patients, including 30 (56%) males and 24 (44%) females with a mean age of 28.3±8.4 years (minimum-maximum: 18-46 years), were included in this study. Of the 54 patients, 46 had homozygous sickle cell anemia (HbSS) and 8 had sickle/β-thalassemia (HbS/β^+^-thalassemia). We found that plasma native thiol (-SH) and total thiol levels (-SH + -S-S-) were significantly lower in SCD patients (respectively 284±86.3 µmol/L and 313±89.3 µmol/L) compared to controls (respectively 417±54.2 µmol/L and 462±58.74 µmol/L). However, plasma disulfide (-S-S-) levels were lower in SCD patients compared to controls (respectively 14.6±7.0 µmol/L and 22.7±11.3 µmol/L). In addition, plasma IMA levels of SCD patients were higher than those of controls (respectively 13.6±3.1 g/L and 8.4±1.6 g/L) (Table 1) and negatively correlated with plasma native thiol (r=-0.730; p=0.0001) and total thiol (r=-0.729; p=0.0001) (Table 2).

Although plasma albumin levels were within the normal reference minimum-maximum (3.4-5.4 g/dL) in the healthy controls and SCD patients, levels were found to be significantly decreased in SCD patients. Albumin levels strongly correlated with both plasma native (r=0.853, p=0.0001) and total thiols (r=0.866, p=0.0001) (Table 2). However, after adjusting disulfide levels according to the albumin concentrations, no significant difference was observed between the patient and control groups.

Disulfide/native thiol ratios were not significantly different between the two groups before or after correction for albumin concentrations. Our study demonstrated that IMA levels significantly increased in SCD patients compared to healthy controls.

## Discussion

There are no other reports in the literature investigating the plasma IMA levels and thiol/disulfide balance in patients with SCD. These findings provide a context for understanding the role of the dynamic thiol/disulfide balance and its relation with IMA, an indicator of oxidative stress, in SCD patients. 

Our study demonstrated that IMA levels significantly increased and serum native (-SH) and total thiol (-S–S- + -SH) levels decreased in SCD patients. Plasma albumin levels were lower in SCD patients and significantly correlated with plasma native and total thiol levels. The disulfide (-S–S-) levels were lower in SCD patients compared to healthy controls. However, there was no significant difference observed between the study and control groups after adjusting disulfide levels according to albumin concentrations.

It is well known that the repeated polymerization of deoxygenated HbS, under hypoxic conditions, causes hypoxia, increased ROS, and impairment of oxidative balance in patients with SCD [17]. In addition, decreasing antioxidant enzyme activities (i.e. glutathione peroxidase, catalase, and superoxide dismutase) and consumption of non-enzymatic antioxidants (i.e. glutathione) exacerbate the oxidative damage [18,19]. It has been accepted that -SH groups of sulfur-containing proteins are also related to the maintenance of oxidative balance and play a critical role in the prevention of oxidative stress [20]. Oxidation of free cysteine residues (-SH) leads to reversible formation of disulfide bonds (-S-S) between thiols and protein thiol groups and abnormal thiol/disulfide hemostasis [6]. Therefore, dynamic thiol/disulfide homeostasis is being increasingly implicated in many disorders associated with ischemia.

Intracellular thiols mainly consist of reduced glutathione (GSH), a low-molecular-weight thiol (LMWT) [21]. Intra-erythrocyte GSH depletion has been shown in many studies and is linked to increased oxidative stress in SCD patients [22,23,24,25,26,27]. Even though the exact mechanism remains to be elucidated, erythrocyte GSH loss is attributed to a variety of factors including increased GSH consumption, substrate availability (i.e. NADPH), and dysfunctional GSH recycling [28]. In the present study we evaluated plasma total thiols and dynamic thiol/disulfide balance for the first time by a novel method described by Erel and Neselioglu [7] in SCD patients.

We found significantly lower levels of plasma native and total thiol levels in SCD patients compared to healthy controls (Table 1). Plasma albumin levels were also strongly correlated with both plasma native (r=0.853, p=0.0001) and total thiols (r=0.866, p=0.0001) (Table 2). In a previous study, Ateş et al. [29] evaluated serum thiol levels in patients with chronic kidney disease (CKD) and speculated that low serum native and total thiol levels may have resulted from low serum albumin concentration in the CKD patients. In contrast to the intracellular environment, in plasma, total thiols are at lower concentrations than cells and are mainly formed by HSA-SH (~80%) and slightly formed by LMWTs (i.e. cysteine, cysteinylglycine, gamma glutamylcysteine, glutathione, and homocysteine) [30]. Therefore, we can say that low plasma native and total thiols may be attributed to the low albumin levels in SCD patients. However, this significant difference did not change between the study and control groups after normalization of native and total thiol levels according to albumin concentrations. This suggests that lower thiol levels are related to SCD and cannot be explained simply by depletion of albumin levels. Interestingly, for disulfide levels, the difference between the two groups was nonsignificant after normalization with albumin. This suggests that lower disulfide levels (before adjusting for albumin) may be explained by decreased albumin levels in the SCD group. Two possible processes may contribute to this phenomenon. First, HSA-SH, as the most abundant thiol in plasma, has an antioxidant role due to its cysteine thiols, which react with ROS [30]. In hypoxic conditions, oxidation of thiols with oxidants leads mainly to sulfenic acid (HAS-SOH) formation, which is an oxidized form of albumin. Sulfenic acid can form reversible disulfides or, in the presence of excess oxidants, it can be further oxidized to sulfinic (RSO_2_H) and sulfonic acids (RSO_3_H), which are irreversible processes. These oxidized forms of albumin have relatively short half-lives and are swept from the circulation by liver cells [30,31]. We suggest that native thiols may change into sulfenic acid forms of albumin, rather than disulfide forms, and be picked up by the liver continuously. Second, glutathione and other LMWTs are necessary for the reduction of oxidized thiol groups. It has been shown that they have relatively low concentrations both in erythrocytes and plasma [30,32]. We can say that the low availability of glutathione and other LMWTs can limit protein mixed-disulfide formation.

IMA (or the albumin cobalt binding test) is used as a novel and sensitive marker of ischemia and oxidative stress mainly for myocardial infarction and various diseases related to ischemia, including peripheral vascular disease, skeletal muscle ischemia, and diabetes mellitus [33,34,35,36]. SCD causes several harmful conditions including sickling, vaso-occlusion, and ischemia-reperfusion injury, which involve the generation of ROS and impairment of oxidative balance. Many studies have shown relationships between oxidative stress parameters and SCD or other related complications such as acute chest syndrome [37] and pulmonary hypertension [38]. In the present study we showed for the first time that IMA levels were significantly higher in SCD patients. Increased IMA levels can be explained by increased ROS production in SCD patients. These data indicate that increased oxidative stress and ROS production in SCD can play a major role in decreased body thiol reserves and cause increased IMA levels.

### Study Limitations

The limitation of our study is that it is a cross-sectional study. Further prospective studies are needed to compare the thiol balance and IMA levels in patients with vaso-occlusive crises and in the steady-state condition.

## Conclusion

Our results support the idea that decreased plasma native and total thiol levels are related to increased oxidative stress. Impaired thiol balance plays an important role in the pathogenesis of SCD. Disulfide levels are also important in evaluating thiol hemostasis, but these levels should be adjusted according to albumin concentrations because unadjusted disulfide levels might give misleading results in patients with low albumin levels. In addition, IMA levels are increased by excessive ROS production and measuring IMA as a marker in plasma samples provides an indirect and quick reflection of the oxidative damage in SCD patients.

## Figures and Tables

**Table 1:**
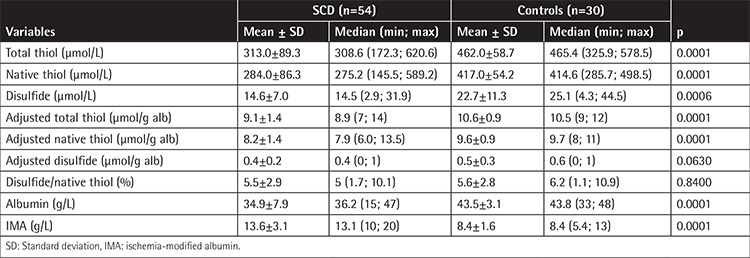
Comparison of thiol/disulfide homeostasis parameters and ischemia-modified albumin levels of the study and control groups.

**Table 2:**
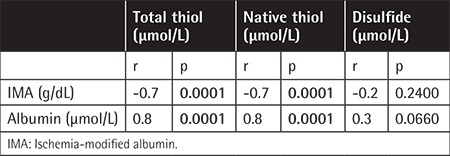
Correlations among plasma ischemia-modified albumin, albumin levels, and thiol/disulfide homeostasis parameters in sickle cell disease patients.
